# Dynamics of Insulin Secretion from EndoC-*β*H1 *β*-Cell Pseudoislets in Response to Glucose and Other Nutrient and Nonnutrient Secretagogues

**DOI:** 10.1155/2017/2309630

**Published:** 2017-10-19

**Authors:** Hiroki Teraoku, Sigurd Lenzen

**Affiliations:** Institute of Experimental Diabetes Research, Hannover Medical School, 30623 Hannover, Germany

## Abstract

The dynamics of insulin secretion were characterized in response to a variety of physiological and pharmacological stimulators and other compounds in perifused pseudoislets generated from cells of the EndoC-*β*H1 *β*-cell line. Perifusion of EndoC-*β*H1 pseudoislets with the physiological stimulus glucose (16.7 mM) induced sustained insulin secretion, which was inhibited by mannoheptulose. The adenylate cyclase activators IBMX and forskolin strongly potentiated this secretion. Glibenclamide, a Kir 6.2 potassium channel blocker, and Bay K 8644, an opener of the voltage-sensitive Ca^2+^ channel, also potentiated glucose-induced insulin secretion. The dynamics of insulin secretion from EndoC-*β*H1 pseudoislets were characterized by an insulin secretory response to glucose starting within 1-2 min and passing over without interruption into a sustained phase of insulin release for the whole stimulation period. This lack of a transient decline between the first and the second phases of insulin release is an indication for a quick supply of insulin secretory granules from the reserve pool to the docking sites below the plasma membrane. Thereby, new secretory granules are directly made available for sustained exocytosis of insulin in EndoC-*β*H1 *β*-cells. The study shows that EndoC-*β*H1 *β*-cell pseudoislets are well suited for kinetic analyses of insulin secretion.

## 1. Introduction

The EndoC-*β*H1 *β*-cell line is the first permanent human *β*-cell line which has been shown to exhibit physiological characteristics comparable to those of primary human *β*-cells [1]. Insulin release experiments in response to a variety of stimulators and other compounds have been performed in incubation studies with the EndoC-*β*H1 *β*-cell line [1–3]. However, kinetic insulin secretion studies have not been performed so far.

These kinetic studies allow the characterization not only of the biphasic secretory pattern of the main physiological stimulus glucose [4–6] but also of the patterns of other physiological and pharmacological stimuli. In particular, it was the aim to distinguish between the first phase of insulin secretion during the initial few minutes of exposure to the secretory stimulus [4–6] from the readily releasable insulin secretory granule pool [7] and the subsequent second phase of secretion [5, 6] from the reserve pool [7].

In the present study, we have generated pseudoislets [8, 9] from these EndoC-*β*H1 *β*-cells [10, 11], which are ideally suited for the performance of kinetic analyses of insulin secretion in perifusion studies [12]. They compare nicely in their insulin secretory responsiveness to isolated rodent and human pancreatic islets. Pseudoislets exhibit much better insulin secretory responsiveness to stimulation than single cells [8, 9].

## 2. Materials and Methods

### 2.1. Chemicals

Tissue culture materials were obtained from Invitrogen, Karlsruhe, Germany. The following chemicals were used in the experiments: d-glucose, l-leucine, l-glutamine, pyruvate, l-lactate, IBMX, and forskolin (Sigma-Aldrich, Taufkirchen, Germany), mannoheptulose (Bujno Synthesis, Warsaw, Poland), glibenclamide (Santa Cruz Biotechnology, Dallas, USA), and Bay K 8644 (Alomone Labs, Jerusalem, Israel). All other reagents were from Merck.

### 2.2. Human EndoC-*β*H1 *β*-Cell Culture and Pseudoislet Formation

EndoC-*β*H1 *β*-cells (ENDOCELLS SARL, Paris, France; http://www.endocells.com/) were cultured, and pseudoislets (PIs) were generated and cultured under the same conditions as described previously [10, 11] following the same principles as those for generation of pseudoislets from insulin secretory cell lines of rodent origin [8, 9].

### 2.3. Perifusion and Insulin Secretion

EndoC-*β*H1 *β*-cell pseudoislets were washed in Krebs-Ringer buffer. Thereafter, 100 pseudoislets were perifused in a specially designed chamber [13]. Perifusion was performed at a flow rate of 1 ml/min at 37°C with 95% O_2_ and 5% CO_2_ with bicarbonate-buffered Krebs-Ringer solution containing 0.1% albumin [5] and supplemented with glucose and the other test compounds as indicated in the figures. Perifusion medium was collected at 0.5, 1, or 5 min intervals, and the insulin released into the perifusion medium was determined by radioimmunoassay using I^125^-labelled pig insulin, with the bound insulin separated by polyethylene glycol 6000 and with human insulin as a standard.

## 3. Results

**Figure 1:**
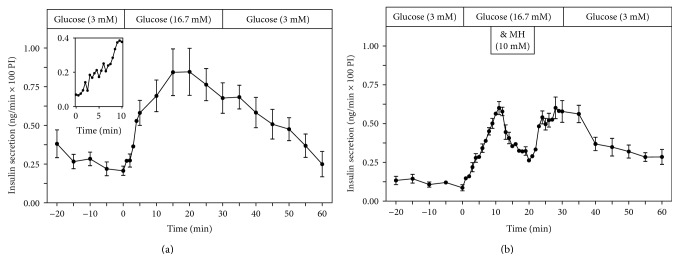
Kinetic profiles of insulin secretion from perifused EndoC-*β*H1 *β*-cell pseudoislets in response to a 30 min stimulation with glucose (16.7 mM) (a) (*n* = 7) and to the inhibition of glucose-induced (16.7 mM) insulin secretion by mannoheptulose (10 mM) (11–20 min) (b) (*n* = 4). After a 20 min preperifusion phase with basal glucose (3 mM), a 30 min stimulation period with high glucose (16.7 mM) and thereafter a return of the perifusion to basal glucose medium (3 mM) again for another 30 min are depicted. The inset in (a) shows a magnification of the initial 10 min glucose (16.7 mM) stimulation period of the perifusion. Shown are means ± SEM of insulin release rates expressed as ng/min and per 100 pseudoislets (PI).

Glucose (16.7 mM) stimulated insulin secretion from perifused EndoC-*β*H1 *β*-cell pseudoislets (Figure 1(a)). When the perifusion medium was changed from 3 mM to 16.7 mM glucose, there was an immediate increase of the insulin secretory rate, which reached a maximum within 10 min and remained high for the whole 30 min stimulation period (Figure 1(a)). Switching the incubation medium back to a basal glucose concentration of 3 mM resulted in a gradual decrease of the insulin secretory rate back to basal rates (Figure 1(a)).

When mannoheptulose (10 mM), a specific inhibitor of glucose-induced insulin secretion, was added during the glucose (16.7 mM) stimulation period for 10 min (from min 11–20), the insulin secretory rate decreased immediately (Figure 1(b)). Upon removal of mannoheptulose from the incubation medium, the glucose-induced (16.7 mM) insulin secretory rate increased immediately again to preinhibitory levels (Figure 1(b)).

The inset in Figure 1(b) with insulin measured at 30 sec intervals in the perifusion medium documents the start of the increased insulin release rate within the 2nd min of exposure to 16.7 mM glucose.

**Figure 2:**
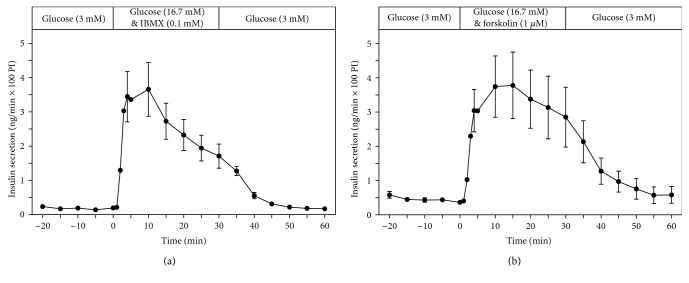
Kinetic profiles of insulin secretion from perifused EndoC-*β*H1 *β*-cell pseudoislets in response to a 30 min stimulation with glucose (16.7 mM) (a) (*n* = 7) in the presence of adenylate cyclase activators IBMX (0.1 mM) (a) (*n* = 5) and forskolin (1 *μ*M) (b) (*n* = 4). After a 20 min preperifusion phase with basal glucose (3 mM), a 30 min stimulation period and thereafter a return of the perifusion to basal glucose medium for another 30 min are depicted. Shown are means ± SEM of insulin release rates expressed as ng/min and per 100 pseudoislets (PI).

The adenylate cyclase activators IBMX (0.1 mM) and forskolin (1 *μ*M) strongly potentiated, by a factor of around 4, glucose-induced insulin secretion (16.7 mM) (Figures 2(a) and 2(b)).

**Figure 3:**
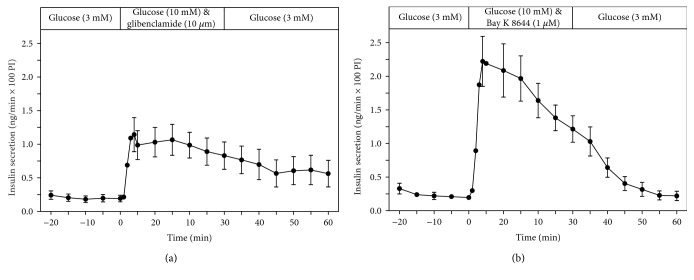
Kinetic profiles of insulin secretion from perifused EndoC-*β*H1 *β*-cell pseudoislets in response to a 30 min stimulation with glibenclamide (10 *μ*M) (a) (*n* = 5) as well as with Bay K 8644 (1 *μ*M) (b) (*n* = 5) in the presence of glucose (10 mM) (a) (*n* = 7). After a 20 min preperifusion phase with basal glucose (3 mM), a 30 min stimulation period and thereafter a return of the perifusion to basal glucose medium for another 30 min are depicted. Shown are means ± SEM of insulin release rates expressed as ng/min and per 100 pseudoislets (PI).

The K_ATP_ channel blocker glibenclamide (10 *μ*M), a sulfonylurea drug, as well as the Ca^2+^ channel activator Bay K 8644 (1 *μ*M) stimulated insulin secretion in the presence of glucose (10 mM) (Figures 3(a) and 3(b)).

**Figure 4:**
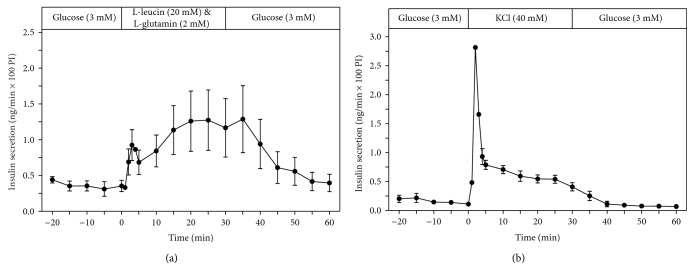
Kinetic profiles of insulin secretion from perifused EndoC-*β*H1 *β*-cell pseudoislets in response to a 30 min stimulation with leucine (20 mM) in the presence of glutamine (2 mM) (a) (*n* = 4) as well as with KCl (40 mM) (b) (*n* = 4). After a 20 min preperifusion phase with basal glucose (3 mM), a 30 min stimulation period and thereafter a return of the perifusion to basal glucose medium for another 30 min are depicted. Shown are means ± SEM of insulin release rates expressed as ng/min and per 100 pseudoislets (PI).

The nutrient insulin secretagogue leucine (20 mM), in the presence of glutamine (2 mM), also stimulated sustained insulin secretion (Figure 4(a)). KCl (40 mM), which depolarises the plasma membrane, also stimulated insulin secretion (Figure 4(b)). However, the insulin secretory response to KCl was transient, with a short initial insulin secretory peak, followed by a low insulin release rate for the rest of the 30 min period of KCl exposure (Figure 4(b)).

**Figure 5:**
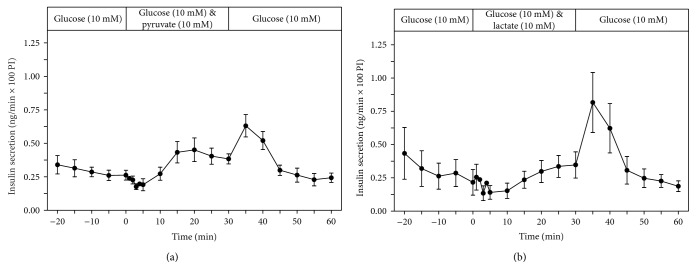
Kinetic profiles of insulin secretion from perifused EndoC-*β*H1 *β*-cell pseudoislets in response to a 30 min exposure to pyruvate (10 mM) (a) (*n* = 4) or to lactate (b) (*n* = 5), each in the presence of glucose (10 mM). After a 20 min preperifusion phase with basal glucose (3 mM), a 30 min stimulation period and thereafter a return of the perifusion to 10 mM glucose medium for another 30 min are depicted. Shown are means ± SEM of insulin release rates expressed as ng/min and per 100 pseudoislets (PI).

In the presence of 10 mM glucose, pyruvate (10 mM) addition caused a minimal increase of the insulin secretory rate and lactate (10 mM) had no effect (Figures 5(a) and 5(b)). After removal of pyruvate from the perfusion medium with glucose (10 mM), a small so-called “off effect” was observed (Figure 5(a)). In the case of the removal of lactate from the perfusion medium, this “off effect” was more pronounced (Figure 5(b)). Such “off effects” after removal of a test compound are an indication of an antagonistic inhibitory effect on the stimulatory effect of glucose.

In Table 1, the results of a quantitative evaluation and statistical analysis of the insulin secretion rates from the perifused EndoC-*β*H1 pseudoislets in response to stimulation with different secretagogues as depicted graphically in Figures 1–5 are presented. With the exception of pyruvate and lactate, all test compounds induced a significant stimulation of insulin secretion when compared to the preperifusion phase with basal medium (Table 1).

## 4. Discussion

A few years ago, Ravassard and colleagues published the first description of the novel human *β*-cell line EndoC-*β*H1, created by genetic engineering [2, 14]. Meanwhile, a number of publications characterizing the physiological features of the human EndoC-*β*H1 *β*-cell line have shown that this permanent *β*-cell line expresses all the crucial biological structures on the gene and protein level which constitute the phenotype of a typical human pancreatic *β*-cell [1, 3]. From these studies, it could be concluded that these *β*-cells react to a wide range of stimulators and modulators of insulin secretion. However, so far no information is available upon the kinetic characteristics of the insulin secretory response. This information is provided in the present study. The results document the same marked insulin secretory responsiveness to stimulation (Figures 1–5; Table 1) as primary rodent and human *β*-cells.

Perifusion of EndoC-*β*H1 *β*-cell pseudoislets with a stimulatory glucose concentration (16.7 mM) induced a sustained insulin secretory response, which was reversible upon return to a basal low glucose concentration. The specificity of the glucose-induced secretory response was documented by the inhibition with the glucokinase inhibitor mannoheptulose, the classical selective inhibitor of glucose-induced insulin secretion [1].

Insulin secretion from EndoC-*β*H1 pseudoislets started within 1-2 min upon an increase of the glucose concentration from 3 mM to a stimulatory glucose concentration of 16.7 mM, as also known from rodent islets [4]. Thereafter, a gradual increase of the secretory rate was observed; however, without a distinct transient, first phase of insulin release clearly separated from a second phase by a nadir as it is seen in rat islets with a progressively increasing second phase of secretion [5, 6] or with a sustained flat second phase of insulin secretion in mouse islets [5, 6, 15]. A comparison with the results of kinetic perifusion studies with isolated human pancreatic islets [16, 17] documents that the secretory response to a glucose stimulus and the return of the secretory rate upon exposure to a basal glucose concentration are comparable to those described here for perifused EndoC-*β*H1 pseudoislets.

This lack of a distinct separate early phase of insulin from perifused human pancreatic islets [16, 17] as well as from perifused human EndoC-*β*H1 pseudoislets can be interpreted as an indication of a quick replenishment of insulin secretory granules from the reserve granule pool, before the rapidly releasable pool at the docking sites below the plasma membrane is depleted. Thus, there appears to prevail a situation in the human *β*-cell, where at variance from rat and in particular from mouse *β*-cells, which is traditionally the preferred species for exocytosis studies in *β*-cells, provision of new secretory granules from the reserve pool to the rapidly releasable pool at the submembrane space is not retarded.

Also in stimulation experiments of EndoC-*β*H1 pseudoislets with other insulin secretagogues at most, a small transient first phase of insulin secretion was observed, which quickly passed over without a clear nadir into a sustained high second phase of release.

Adenylate cyclase activators such as IBMX and forskolin strongly potentiated glucose-induced insulin secretion by several folds from perifused EndoC-*β*H1 pseudoislets, which is due to a stimulation of cAMP generation [6, 18]. This shows that EndoC-*β*H1 pseudoislets are responsive to potentiators which act via the second messenger cAMP, as has been shown before for EndoC-*β*H1 *β*-cells in response to GLP-1 [2], which also acts via the second messenger cAMP [19].

A strong potentiation of insulin secretion was also observed by the sulfonylurea drug glibenclamide, a Kir 6.2 potassium channel blocker that exerts its effect via interaction with the sulfonylurea receptor SUR1 and by Bay K 8644, a voltage-sensitive Ca^2+^ channel activator, which mediates its potentiating effect on insulin secretion through opening of this channel. This is in line with earlier observations in incubated EndoC-*β*H1 *β*-cells [1], and it supports the earlier conclusion that the EndoC-*β*H1 *β*-cells are like primary *β*-cells, equipped with all crucial structures (see Figure 4 in [1]) for initiation and maintenance of the typical kinetic insulin secretory responses to the various stimuli.

Depolarisation of the plasma membrane upon perifusion with KCl induced only a transient short-lived insulin secretory response from the perifused EndoC-*β*H1 pseudoislets, indicating that a sustained kinetic secretory response, as it is seen in response to stimulation with the nutrient insulin secretagogue glucose, requires a constant fuel supply to the *β*-cell.

The EndoC-*β*H1 pseudoislets mirror exactly the situation prevailing in primary rat islets [18], where pyruvate is a weak potentiator of glucose-induced insulin release and lactate, also a metabolite of glycolytic origin, which completely lacks even a minimal potentiating effect on insulin secretion [18]. This confirms an earlier observation with incubated EndoC-*β*H1 *β*-cells [1] but is at variance from another report [3]. This situation in the EndoC-*β*H1 *β*-cells is different from clones of the permanent rat INS1 tissue culture cell line, which differ from primary *β*-cells through a nonphysiological insulin release in response to lactate [20, 21].

The lack of an insulin secretory potency of lactate in EndoC-*β*H1 *β*-cells is an indicator for a well-differentiated status of this human *β*-cell line and can be interpreted as a favourable feature of the EndoC-*β*H1 *β*-cell, in contrast to rat insulin-secreting cell lines of tumorigenic origin [20, 21], making the EndoC-*β*H1 *β*-cell a better surrogate for primary *β*-cell metabolic studies than permanent rat *β*-cell lines.

## 5. Conclusion

Pseudoislets prepared from *β*-cells of the human EndoC-*β*H1 cell line are thus very well suited for analyses of the dynamics of insulin secretion. They mirror the situation in primary pancreatic islets very well and can act as a suitable surrogate for primary human pancreatic islets and primary human *β*-cells in experimental studies.

## Figures and Tables

**Table 1:** Quantitative evaluation and statistical analysis of the insulin secretion rates from perifused EndoC-*β*H1 pseudoislets in response to stimulation with different secretagogues.

		Preperifusion	Perifusion		
Stimulation by	Figure	Insulin secretion (ng/min)	Insulin secretion (ng/min)	*N*	*p* value
A	B	C	D	E	F
Glucose (16.7 mM)	1(a)	0.244 ± 0.037	0.705 ± 0.104	7	<0.01
Glucose (16.7 mM) & IBMX (0.1 mM)	2(a)	0.176 ± 0.030	2.439 ± 0.474	4	<0.01
Glucose (16.7 mM) & forskolin (1 *μ*M)	2(b)	0.418 ± 0.065	3.138 ± 0.792	5	<0.01
Glucose (10 mM) & glibenclamide (10 *μ*M)	3(a)	0.194 ± 0.049	0.938 ± 0.203	5	<0.01
Glucose (10 mM) & Bay K 8644 (1 *μ*M)	3(b)	0.206 ± 0.036	1.631 ± 0.276	5	<0.01
Leucine (20 mM) & glutamine (2 mM)	4(a)	0.275 ± 0.062	0.849 ± 0.262	4	<0.05
Glucose (10 mM) & KCl (40 mM)	4(b)	0.155 ± 0.041	0.681 ± 0.086	3	<0.01
Glucose (10 mM) & pyruvate (10 mM)	5(a)	0.281 ± 0.040	0.358 ± 0.051	4	Ns
Glucose (10 mM) & lactate (10 mM)	5(b)	0.257 ± 0.105	0.254 ± 0.073	5	Ns

For the different experimental conditions (A) as depicted in Figures 1–5 (B), the rates of insulin secretion for 100 perifused islets (expressed as ng/min) were calculated for the preperifusion phase (20 min) (C) and the perifusion phase (30 min) (D) with the numbers of experiments (E) and the *p* values calculated with the unpaired *t*-test (F). Ns: no significance.
